# Astilbin from* Smilax glabra* Roxb. Attenuates Inflammatory Responses in Complete Freund's Adjuvant-Induced Arthritis Rats

**DOI:** 10.1155/2017/8246420

**Published:** 2017-08-22

**Authors:** Lisha Dong, Jinqiu Zhu, Hongzhi Du, Heng Nong, Xicheng He, Xiaoyu Chen

**Affiliations:** ^1^School of Pharmacy, Guiyang College of Traditional Chinese Medicine, Guiyang 550002, China; ^2^Center of Miao Medicine Engineering Technology, Guiyang 550002, China; ^3^School of Public Health, Key Laboratory of Public Health Safety, Ministry of Education, Fudan University, Shanghai 200032, China; ^4^Department of Epidemiology and Environmental Health, The State University of New York, Buffalo, NY, USA; ^5^School of Chinese Medicine, Hong Kong Baptist University, Kowloon Tong, Hong Kong

## Abstract

Astilbin, a flavonoid compound, was isolated from the rhizome of* Smilax glabra* Roxb. (with red cross-section) grown in Guizhou Province, China. We accessed its effect and potential mechanism on attenuation of the inflammatory response in CFA-induced AA rats. Our results showed that daily oral administration of astilbin at 5.3 mg/kg reduced joint damage in the hind paw of AA rats. Accordingly, astilbin exhibited remarkable inhibitory effects on TNF-*α*, IL-1*β*, and IL-6 mRNA expression. Significant decrease of serum cytokine levels of TNF-*α*, IL-1*β*, and IL-6 was also observed in astilbin-treated AA rats compared to the vehicle-treated AA rats. The reduced expression of these cytokines was associated with protein activity suppression of three key molecular targets in the pathogenesis of RA, including IKK*β*, NF-*κ*B p65 subunit, and TLR adaptor MyD88. Furthermore, the therapeutic effects of astilbin on the inhibition of cytokines production as well as the reduction of inflammatory response in AA rats are close to a commonly used antirheumatic drug, leflunomide. Collectively, our data suggest that the action mechanism of astilbin, as an anti-inflammatory agent for RA treatment, is associated with modulating the production of proinflammatory cytokines and inhibiting the expression of key elements in NF-*κ*B signaling pathway mediated by TLR.

## 1. Introduction 

Rheumatoid arthritis (RA) is an autoimmune disease characterized by chronic inflammation in joints, associated with inflammatory infiltration of the synovia, and cartilage/bone destruction. It affects nearly 1% of the population worldwide [1]. Pathogenic investigations of bone erosion in RA have reflected the primary involvement of cellular and soluble mediators of the immune system, such as cytokines, adhesion molecules, and prostaglandins [2]. The main triggers of bone erosion are synovitis, including the production of proinflammatory cytokines and receptor activator of nuclear factor kappa B ligand, which cause the destruction of cartilage, bone, and periarticular structures [3]. Inflamed synovial tissues are infiltrated by monocyte/macrophage, synovial fibroblast, T cells, and B cells, which are considered as key players in the mammalian immune system [4]. In response to such immune/inflammatory stimuli, proinflammatory cytokines, including but not limited to tumor necrosis factor-*α* (TNF-*α*), interleukin-1 (IL-1), and IL-6, are subsequently released by these cells [5]. An increasing body of data has shown that cytokines are implicated in each phase of progressive joint destruction, by autoimmunity promotion, maintenance of chronic inflammatory synovitis, and driving the destruction of adjacent joint tissue [6]. Due to their important roles in the pathogenesis of RA, cytokines have provided a whole new range of targets for drug development, and many of them are already being targeted in the clinic for clinical intervention [7]. Accordingly, new therapies targeting the modulation of cytokine release during RA therapy have been investigated [8]. In addition, emerging evidence suggests that transcription factor nuclear factor kappa B (NF-*κ*B) is essential for the expression of inflammatory cytokines. Toll-like receptors (TLRs), as major activators of the NF-*κ*B pathway, are also involved in triggering the inflammatory and joint destructive process in RA [7]. Therefore, blockade of NF-*κ*B signaling serves as a potential therapeutic strategy for RA treatment [9].

*Smilax glabra *Roxb. (SRG, genus:* Smilax*), the dried rhizome of Sarsaparilla, also called Tu-Fuling in Chinese medicine, is commonly used as the complementary therapy in the Orient for various chronic diseases, including RA, diabetes, liver deficiency, coronary heart, and syphilis [10]. It is officially included in the Chinese Pharmacopeia and has been investigated for its antivirus, antioxidant, anticancer, and anti-inflammation properties [11–13]. SRG is classified into two categories based on its cross-sectional color, red SGR or white SGR, and the investigation of their chemical composition indicates significant difference in the quality correlating to their color metrics [10, 14]. The aqueous SGR extract is known to modulate cellular immune responses [15]. Our previous studies have shown that the anti-inflammatory effects of red-SRG, a species from Guizhou Province of Southwestern China (GZ-SGR), are better than white SGR for reducing chronic inflammation in the adjuvant arthritis (AA) rat model, suggesting the therapeutic potential of GZ-SGR in the treatment of chronic autoimmune disease [14]. Astilbin, 3,3,4′,5,7-pentahydroxyflavanone 3–6[-deoxy-([alpha]-L-mannopyranoside)], is one of the major active components in the rhizome of SGR. This flavonoid shows multiple pharmacological functions, such as migration of ear contact dermatitis and liver injury induced by delayed-type hypersensitivity associated with selective immunosuppression of activated T lymphocytes or dysfunction of liver-infiltrating cells [13, 16, 17]. Nevertheless, the direct role of astilbin, isolated from GZ-SGR extract, in the treatment of inflammatory disease such as RA, is still unclear and the mechanistic basis is lacking. In the present study, we used the CFA-induced arthritic rat model to evaluate the therapeutic effects of astilbin on the prevention of joint damage associated with RA and further explored the possible molecular mechanism.

## 2. Material and Methods

### 2.1. Plant Material and Astilbin Isolation

**Figure 1:**
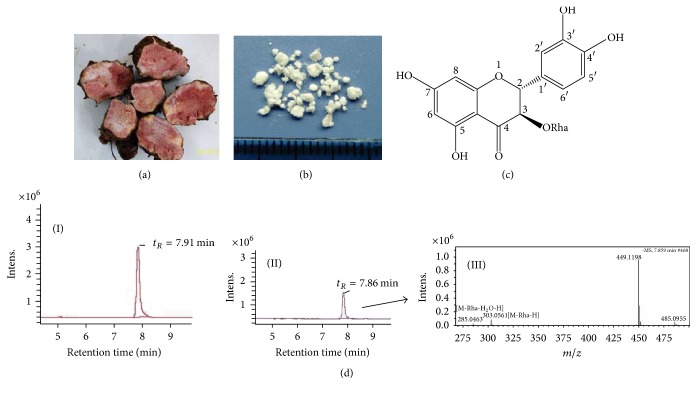
*Astilbin isolation and identification.* (a) Rhizome cross-section of Guizhou* Smilax glabra* Roxb. (GZ-SGR). (b) Crystallized astilbin powder isolated from GZ-SGR. (c) Chemical structure of astilbin. (d) UPLC chromatogram of isolated astilbin from GZ-SGR (II), in comparison with standard astilbin (I). Mass spectrum for the peak of GZ-SGR astilbin is shown in (III).

GZ-SGR was obtained from Shuitian Town of Wudang County in Guizhou Province, China. The voucher specimens were deposited at Key Pharmacognosy Laboratory, Guiyang Collage of Traditional Chinese Medicine (Figure 1(a)). The procedures for preparation of aqueous extract from the rhizome of GZ-SGR and the methods for astilbin isolation were described previously with a few modifications [18, 19]. Briefly, the dried and cut rhizome of GZ-SGR was extracted with 60% ethanol under reflux (2 h each, ×3). After removal of the solvent by evaporation, the ethanol extract was suspended in water and successively partitioned with ethyl acetate (EtOAc). A portion of EtOAc-soluble fraction (70 g) was subjected to a silica gel column and eluted with a gradient of chloroform-EtOAc-MeOH to give 3 fractions. Astilbin detected in the second fraction was further purified via preparative high performance liquid chromatography (HPLC). Isolated astilbin showed the purity more than 99% analysis (Figure 1(b)), followed by characterization using an ultra-performance liquid chromatography/quadrupole time-of-flight mass spectrometry (UPLC/QTOF MS) system [10]. The UPLC chromatogram and mass spectrum of isolated astilbin from GZ-SGR were shown in Figure 1(d). Astilbin (Cat# 30-2007) standard was purchased from Shanghai R&D Center for Standardization of Traditional Chinese Medicines (Shanghai, China). Leflunomide (LEF) was obtained from Changzheng-Cinkate Pharmaceutical Co., Ltd. (Cat# 140304, Suzhou, China).

### 2.2. Rats and Treatment

Male Sprague-Dawley rats weighing 180 ± 20 g were purchased from the Chongqing Animal Center of the Third Military Medical University, China (certification no. SCXK-YU-20120005). All rats were provided a standard diet and housed in an approved facility with climate control and a 12 h light/12 h dark cycle. This study was performed in strict accordance with the recommendations in the Guide for the Care and Use of Laboratory Animals of the National Institutes of Health. The protocol was approved by the local animal ethics committee at the Third Military Medical University, China. All surgery was performed under urethane anesthesia with effort to minimize suffering. Forty rats were randomly grouped into control and three treatment groups (*n* = 10 in each): model, astilbin (GZ-SRG), and LEF. The vehicle alone (0.5% CMC-Na) was administered via gavage to the control group. Adjuvant arthritis was elicited by injecting complete Freund's adjuvant (CFA, 0.1 ml, 10 mg/ml) into the base of the tail every day for 7 days. In the following treatment regimen, rats in astilbin and LEF groups were orally administered with astilbin in 0.5% CMC-Na at 5.7 mg/kg or 2.3 mg/kg/day for 21 days, respectively. In parallel, the vehicle was administered via oral gavage to the model group and control group. At the end of treatment, rats were sacrificed and radiographs of tibiotarsal joint of the hind paw were taken with an X-ray instrument (40 kV, 100 mA, 6/100 s) and X-OMAT TL films.

### 2.3. Histopathology Evaluation

Synovial tissues with patella but without menisci were obtained from the knee joint of rats. The specimens were postfixed overnight in buffered 10% formalin, dehydrated through a series of ethanol, and embedded in paraffin wax. They were serially sectioned onto microscope slides at a thickness of 5 *μ*m and then deparaffinized, stained with hematoxylin and eosin, and evaluated for morphological changes and cellular infiltration, as previously described [20].

### 2.4. Quantitative Real-Time PCR

Total RNA from synovial tissues of rats was extracted using TRIzol Reagent (Life Tech., Carlsbad, CA). mRNAs were reverse-transcribed using a PrimeScript™ RT Reagent Kit (Takara, Dalian, China). Gene expression of* TNF-α*,* IL-1β*, and* IL6* in rats was measured via quantitative real-time PCR using primers listed in Table 1. Real-time PCR was performed using Bio-Rad CFX96 Touch™ Real-Time PCR Detection System and a SYBR Green Supermix Kit (Bio-Rad Laboratories, Hercules, CA). The PCR efficiency was examined by serially diluting template cDNA and the melting curve data were collected to check the PCR specificity. Results were calculated using the comparative CT (2^−ΔΔCT^) method normalizing to glyceraldehyde 3-phosphate dehydrogenase (GAPDH) expression for each sample.

### 2.5. Analysis of Serum Cytokines

Serum was separated from whole blood samples after centrifugation (2,500 ×g for 10 minutes at 4°C) and stored in −80°C until analysis. Analysis of serum cytokines was measured by PicoKine™ ELISA Kit (BosterBio, Wuhan, China) according to the manufacturer's protocol (rat TNF-*α*, 2401122312; rat IL-1*β*, 11510123312; rat IL-6, 13310123312), as previously described [21].

### 2.6. Western Blot

After the rats were killed, knee synovium of rat was collected, and total proteins were extracted from synovial tissue and quantified by the BCA assay (Cwbiotech, Beijing, China). Protein expression was detected by Western blot. Equal protein amounts were resolved by sodium dodecyl sulfate polyacrylamide gel electrophoresis (SDS-PAGE), transferred onto PVDF membranes, and immunoblotted for MyD88 (Cat# RLT2928, Ruiying Bio, Suzhou, China), p65 (Cat# B0442), and IKK*β* (Cat# B7169, Assay Biotech, USA). Each measured protein was normalized to GAPDH (Cat# CW0100, Cwbiotech, Beijing, China) and quantified using ImageJ software (NIH, Bethesda, USA).

### 2.7. Statistical Analysis

Data are expressed as means ± SD from at least three independent experiments. Statistical significance between groups was measured using one-way analysis of variance (ANOVA) followed by Student's two-tailed* t*-test. Data analysis was achieved with SPSS 20.0 statistical package (Chicago SPSS Inc., IL, USA). Significance was noted in corresponding figure legends (^*∗*^*p* < 0.05; ^*∗∗*^*p* < 0.01; and ^##^*p* < 0.01).

## 3. Results

### 3.1. Treatment of Astilbin Mitigated Joint Damage in CFA-Induced Arthritic Rats

**Figure 2:**
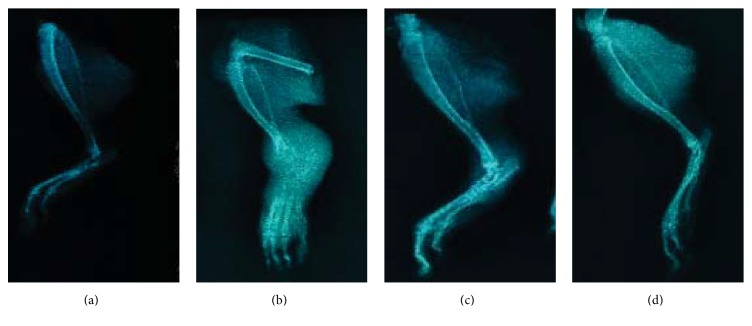
*Radiological assessment of astilbin's effect on tibiotarsal joint destruction in rats with adjuvant-induced arthritis (AA).* ((a)–(d)) Demonstrating typical representative photographs of rat ankle joints. (a) Control group; (b) AA rats group; (c) astilbin-treated AA rats group; (d) LEF-treated AA rats group. The swelling in the hind paw of rats was assessed by soft tissue X-ray examination on day 21 following astilbin or LEF treatment as described in “Materials and Methods.”

**Figure 3:**
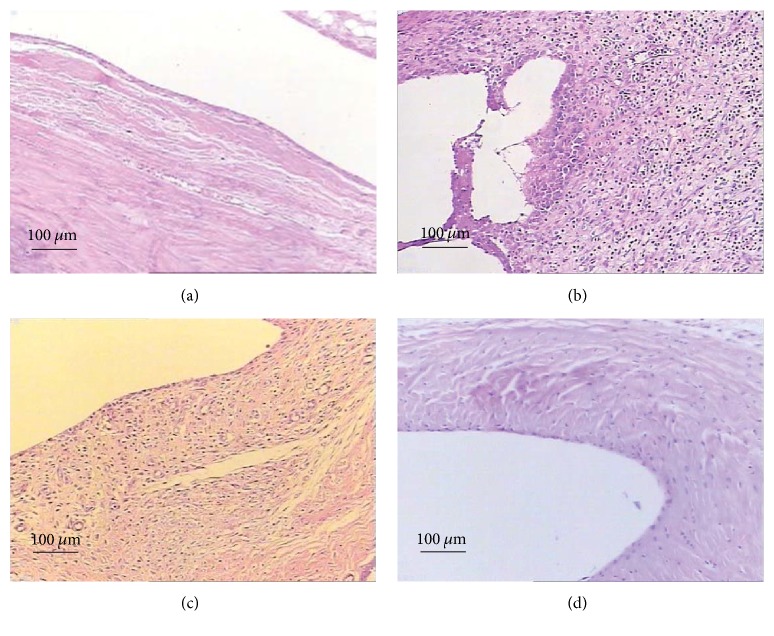
*Photomicrographs of the synovial membrane of the ankle joint in rats*. ((a)–(d)) Demonstrating typical representative H & E stain of rat synovial tissue. (a) Control group; (b) AA rats group; (c) astilbin-treated AA rats group; (d) LEF-treated AA rats group. Magnification, 400x; scale bar = 100 *μ*m.

To examine astilbin's effects on the of RA disease development, we established a CFA-induced arthritis rat model. Under the dose used, no animal died, and body weight gain was comparable to the control group and no obvious behavioral changes were observed (data not shown). CFA injection into the rat tail induced a monoarthritic process characterized by severe radiological joint damage. Seven days after administration of the adjuvant CFA, the signs of joint inflammation became apparent, suggesting that arthritis was clearly developed (Figure 2(b)). Repeated injections of CFA significantly and progressively increased the paw edema (Table 2). Astilbin was then administered once daily by gavage to AA rats at dose level of 5.7 mg/kg/day for 21 days. Examination of the radiographs in astilbin-treated rats revealed a clear lesion decrease that was also observed in LEF-treated rats when compared to CMC-Na treated controls (Figures 2(c) and 2(d)). In the soft X-ray examination, a marked reduction of swelling in the hind paw was seen in the astilbin-treated AA rats. Though cartilage erosion was not prevented completely in this treatment cycle, the antirheumatic effects of astilbin are close to LEF. The decrease of joint lesions was confirmed by histological examination on day 21 after astilbin administration (Figure 3): treatment with astilbin led to a significant inhibition of inflammatory cell infiltration against adjuvant-induced arthritis in rats. In addition, astilbin administered groups showed significant reduction in paw volume when compared with the arthritic model group (Table 2).

### 3.2. Treatment of Astilbin Reduced Inflammatory Cytokine Levels in Adjuvant Arthritic Rats

**Figure 4:**
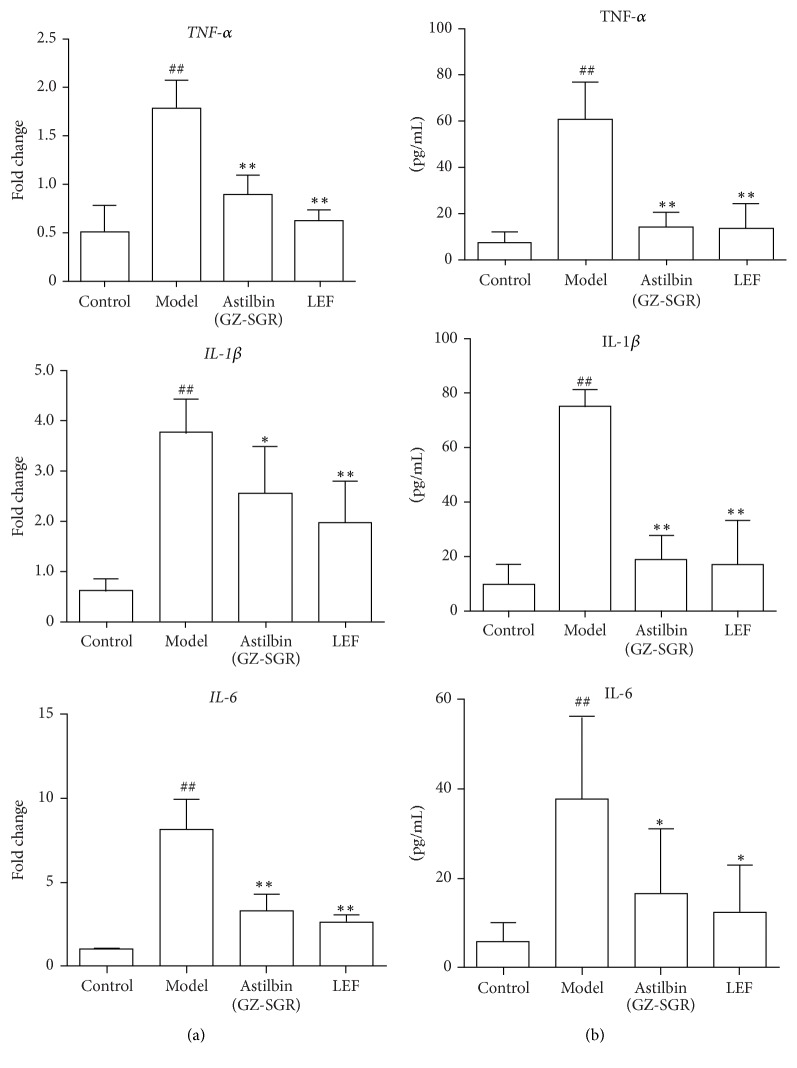
*Astilbin treatment reduced proinflammatory cytokine production.* (a) The mRNA expression of* TNF-α*,* IL-1β*, and* IL-6* in the synovium from rats was examined by real-time PCR. (b) Serum cytokine levels of TNF-*α*, IL-1*β*, and IL-6 in rats were determined by PicoKine ELISA Kit (BosterBio, Wuhan, China). Data shown represent the mean ± SD of three independent experiments, *n* = 10 rats per group as described in Materials and Methods. ^##^*p* < 0.01, versus control group; ^*∗*^*p* < 0.05, ^*∗∗*^*p* < 0.01, versus CFA-induced arthritic rat model group.

To investigate the effects of astilbin on modulating inflammatory response induced by CFA in adjuvant arthritic rats, the proinflammatory cytokines, namely, TNF-*α*, IL-1*β*, and IL-6, were chosen based on their reported association with RA [22]. By quantitative real-time PCR, mRNA levels of* TNF-α*,* IL-1β*, and* IL-6* in synovium of AA rats turned out to be dramatically elevated with arthritis development in our CFA-induced arthritic rat model, while astilbin treatment caused a significant reduction of such cytokine profiles, especially with substantial effects on* TNF-α* and* IL-6* (Figure 4(a)). Remarkably, there is no significant difference between astilbin-treated group and LEF-treated group, suggesting the therapeutic potential of astilbin as an antirheumatic drug for inflammatory modulation. Meanwhile, serum cytokine levels were measured using ELISA assay on day 21 after oral administration of astilbin to AA rats. As shown in Figure 4(b), at dose of 5.7 mg/kg, astilbin significantly reduced serum cytokine levels of TNF-*α*, IL-1*β*, and IL-6, compared to vehicle-treated AA rats. Of note, these cytokines decreased to the levels of those in LEF treatment group. These results clearly showed that astilbin administration attenuated inflammation and inhibited both joint* TNF-α*,* IL-1β*, and* IL-6* expression (mRNA) and serum TNF-*α*, IL-1*β*, and IL-6 levels.

### 3.3. Astilbin Negatively Regulated TLR-Mediated NF-*κ*B Signaling by Targeting MyD88, p65, and IKK*β*

**Figure 5:**
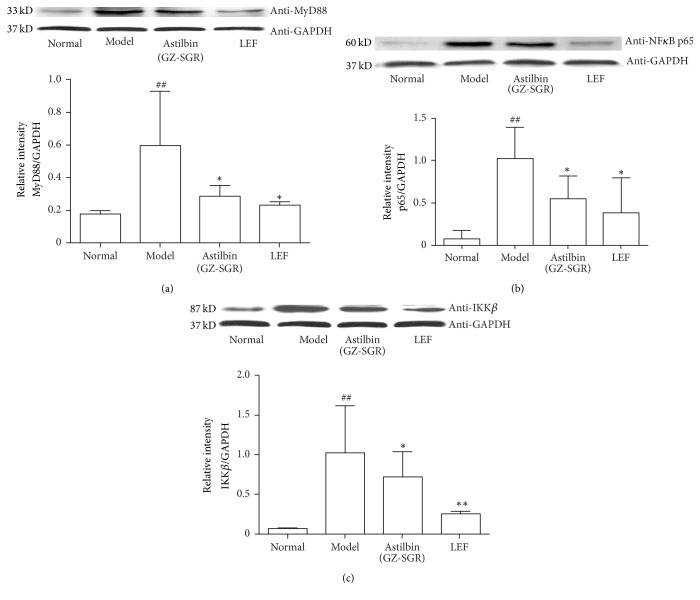
*Astilbin (GZ-SGR) negatively regulated TLRs-mediated NF-κB signaling.* Representative immunoblots and quantitative analysis showed a significant increase protein levels of (a) MyD88, (b) p65, and (c) IKK*β* in CFA-induced AA rats, while astilbin treatment alleviated the increase. Data shown represent the mean ± SD of three independent experiments, *n* = 10 rats per group as described in Materials and Methods. ^##^*p* < 0.01, versus control group; ^*∗*^*p* < 0.05, ^*∗∗*^*p* < 0.01, versus CFA-induced arthritic rat model group.

To determine the mechanisms by which astilbin suppresses adjuvant arthritis in rats, we next examined the effect of astilbin extract from GZ-SRG on TLR-mediated NF-*κ*B signaling, with special focus on the protein expression patterns of MyD88, p65, and IKK*β*. These target molecules were chosen because most TLR use MyD88 as the adaptor protein for downstream signal transduction to NF-*κ*B, while both I*κ*B kinase molecule IKK*β* and p65 subunit are core elements of the NF-*κ*B cascade and serve as pivotal regulators in inflammatory arthritis [23, 24]. As expected, expression levels of protein MyD88, p65, and IKK*β* were all increased in the synovial tissue of AA rats compared to vehicle controls, suggesting that NF-*κ*B activation via TLR is required for inflammatory induction in RA (Figure 5). In contrast, treatment of astilbin resulted in a significant downregulation of Myd88 activity, as shown in Figure 5(a). In addition, the protein levels of NF-*κ*B p65 and IKK*β* were found to be decreased (Figures 5(b) and 5(c)). Accordingly, the levels of MyD88, p65, and IKK*β* were reduced to a similar degree upon LEF treatment in AA rats. These results indicated astilbin's therapeutic potential for the blockade of TLR-mediated NF-*κ*B signaling in the treatment of chronic inflammation.

## 4. Discussion

With the growing interest in herbal medicines for RA treatment, there exists a need for investigation into their safety, efficacy, and mechanism. As a member of the Smilacaceae family, SGR is traditionally used to treat rheumatism, swelling, and pain in China [14]. SGR contains many bioactive components, such as flavonoids, terpenoids, and mannose-binding lectin, which are responsible for its clinical utilization in the treatment of leptospirosis, bacterial dysentery, nephritis, mercury poisoning, and so forth [25, 26]. Previous research has indicated that the aqueous extract from the rhizome of SGR possesses notable immunomodulatory effects for RA treatment by suppressing specific cellular inflammatory activities, while the underlying mechanism has not yet been fully elucidated [15]. Astilbin, a dihydroflavonol derivative isolated from SGR, exhibits multiple pharmacological aspects, including antioxidant, anti-inflammatory, and antidiabetic nephropathy properties [27, 28]. Evidence has shown that astilbin can inhibit contact hypersensitivity through altering the in vivo cytokine profiles of lymphocytes [29], reduce collagen-induced arthritis via the dysfunction of lymphocytes [30], and mitigate disease development in lupus-prone mice by suppressing the functional activated T and B cells [31]. Although the use of SGR in folk medicine to treat arthritis was reported, the antiarthritic effects of its major bioactive compound astilbin have not yet been examined. Moreover, astilbin's direct molecular targets and the underlying mechanisms that explain its anti-inflammatory activities in RA treatment remain to be elucidated. The current study represents the first report of anti-inflammatory effect of astilbin, isolated from GZ-SRG with red cross-section, in CFA-induced arthritic rats.

In our arthritic rat model, the development of arthritis was observed as edema of the hind paw due to severe soft tissue injuries around the joint. Astilbin administered groups showed a significant reduction in paw volume as well as decreased inflammatory cell infiltration when compared with the arthritic model group (Table 2, Figures 2 and 3). It is worth mentioning that the antirheumatic effects of astilbin are pretty close to LEF, an anti-inflammatory agent clinically used to treat RA and other inflammatory conditions [32]. In the present study, repeated administration of astilbin at 5.7 mg/kg/day for 21 days did not result in significant weight loss (data not shown). We next demonstrated that astilbin significantly decreased serum levels of cytokines TNF-*α*, IL-1*β*, and IL-6 after long-term oral injection in AA rats (Figure 4). Meanwhile, the mRNA expression levels of TNF-*α*, IL-1*β*, and IL-6 were greatly downregulated in the synovial tissue from astilbin-treated AA rats, indicative of an inhibition of inflammatory response. Reduction in these cytokine profiles provides a favorable benefit for the migration of joint damage in the rehabilitation management of RA.

As a symmetric peripheral polyarthritis, RA is often associated with chronic inflammation of the synovium of small joints [33]. Although the etiology of RA is not yet fully clear, exaggerated inflammatory mechanisms of cytokines contribute significantly to joint tissue damage in RA [6, 22]. Over the past decade, there have been major advances in the specific immunomodulation of RA as a straightforward therapeutic approach, including the treatments by herbal complementary and alternative medicine (CAM), targeted to molecular messengers important in the immune-pathogenesis of RA [34]. Patients with active RA have increased secretion of multiple cytokines along with macrophage activation, including but not limited to TNF-*α*, IL-1*β*, and IL-6 [22, 35]. These cytokines stimulate synovial fibroblasts and chondrocytes in the nearby articular cartilage to secrete enzymes that degrade proteoglycans and collagen, leading to tissue destruction [22]. It is well established that TNF and IL-1 are the key proinflammatory cytokines in the process of chronic joint inflammation and the concomitant erosive changes in cartilage and bone. Regulation of these cytokines is of crucial importance in the pathogenesis of RA [22]. IL-6 is a pleiotropic cytokine found in abundance in the synovial fluid and serum of subjects suffering from RA, with a correlation to the level of joint destruction [36]. IL-6 can also promote synovitis by upregulating expression of certain chemokines and adhesion molecules. Excess production of IL-6 contributes to inducing hepcidin production and thrombocytosis, which lead to anemia common in active RA [37]. Blocking aforementioned cytokines produces significant improvement in RA and therefore is considered as an important therapeutic goal [38, 39]. Targeting TNF, for example, has shown great efficacy in controlling both the inflammation and structural damage of the joints [7]. New TNF-*α* blockers, IL-1 receptor antagonist, and IL-6 inhibitors are currently being explored for their potential as new immune therapies [39].

**Figure 6:**
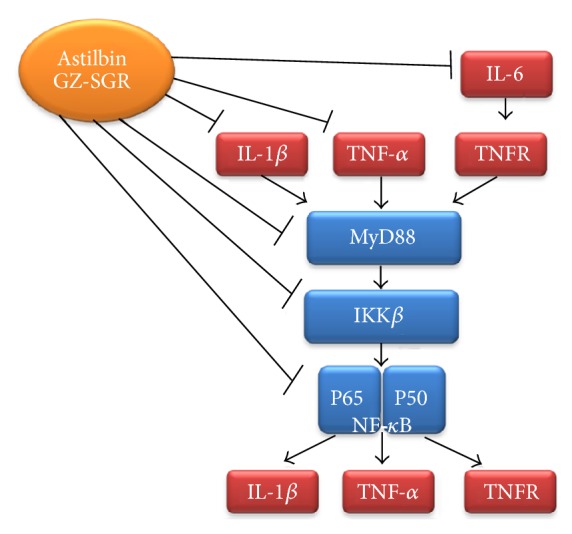
*Schematic diagram of alleviation of rheumatoid arthritis (RA) by astilbin (GZ-SGR).* The canonical pathway NF-*κ*B is involved in the pathogenesis of RA. In the cytoplasm, NF-*κ*B proteins are usually associated with inhibitors of NF-*κ*B (I*κ*Bs). External stimuli, including ligation of receptors of proinflammatory cytokines, activate the TLR adaptor MyD88 and subsequently stimulate the I*κ*B kinase (IKK) molecules, including IKK*β*. IKK phosphorylates I*κ*Bs, leading to their degradation, thereby allowing NF-*κ*B to enter the nucleus and activate genes that contribute to chronic inflammation, such as cytokines, chemokines, and tumor necrosis factor receptors (TNFR). Astilbin, isolated from GZ-SGR, can reduce proinflammatory cytokines production, including TNF-*α*, IL-1*β*, and IL-6, and decrease the protein expression of MyD88, IKK*β*, and p65, the key components that play important roles in TLRs-NF-*κ*B pathway in RA development.

Finally, our results indicated that astilbin had an obvious inhibitory effect on the expression of three key molecular targets, MyD88, p65, and IKK*β*, which are associated with NF-*κ*B signaling and all play important roles in the development of RA. As illustrated in Figure 6, we believe that astilbin's effects on the inflammatory modulation of cytokine production as well as the prevention of RA process are mainly mediated by TLRs-NF-*κ*B pathway. In accordance with the inhibition of the secretion of cytokines TNF-*α*, IL-1, and IL-6, astilbin negatively regulates MyD88, p65, and IKK*β* protein expression, subsequently leading to the inhibition of the NF-*κ*B pathway for the treatment of chronic inflammation associated with RA.

TLRs are a family of integral glycoproteins, well known for their role in the initiation of the inflammatory and immune response [40]. As the potent activators of the NF-*κ*B pathway, TLRs are involved in triggering the inflammatory and joint destructive process in RA [41]. Activation of TLRs results in the rapid expression of inflammatory cytokines including those for proinflammatory mediators including TNF-*α*, IL-1, and IL-6, as well as chemokines, such as IL-8. These mediators thus initiate an immune response that recruits neutrophils, monocytes, and lymphocytes [7]. MyD88 is an adaptor protein shared by all the known TLRs. It is known that TLRs induce cytokine production in response to inflammatory stimuli targeting at MyD88 [42]. The MyD88-dependent pathway also leads to the activation of NF-*κ*B and consequently mediates proinflammatory cytokine gene expression [43]. Furthermore, there is a complex regulatory loop which amplifies and perpetuates inflammatory response. NF-*κ*B is one of the key regulators in this amplifying loop, which sustains chronic inflammation in RA [24]. Proinflammatory stimuli, such as lipopolysaccharide and TNF-*α*, lead to phosphorylation of the inhibitory complex (I*κ*B), subsequently inducing the activation of the NF-*κ*B pathway. IKK*β* is one of the subunits of I*κ*B and has become a particularly appealing target for therapeutic intervention in RA due to its crucial role in the canonical NF-*κ*B pathway activation [44]. Inhibitors of the kinase activity of IKK*β* offer opportunities for RA intervention [45]. p65 (RelA) subunit is one of the most prevalent activated forms of NF-*κ*B family. Expression of activated NF-*κ*B p65 was found abound in the synovial lining layer of RA synovial tissue [46]. siRNA targeting of NF-*κ*B p65 subunit has shown promising effects by suppressing the IL-1*β*/TNF-*α* induced gene expression of cyclooxygenase-2 (COX-2), nitric oxide synthase-2 (NOS-2), and matrix metalloproteinase-9 (MMP-9), which is paralleled with the initiation and progression of cartilage lesions in osteoarthritis [47]. Given these evidences, MyD88, p65, and IKK*β* are all considered as pivotal molecules in the development of chronic inflammation in RA.

In summary, our results demonstrated that, similar to the present immunosuppressant drug leflunomide, astilbin, isolated from GZ-SRG, significantly mitigated RA disease development in CFA-induced arthritic rats by effectively suppressing functionally activated cytokines and the key molecules that are associated with TLRs-NF-*κ*B signaling of the inflammatory response pathway in RA disease process. This potent, orally active, natural immunosuppressant would take advantage to treat the clinic-pathological manifestations of RA.

## 5. Conclusion

In conclusion, astilbin, a natural product isolated from GZ-SGR, showed anti-inflammatory effects through negative cytokine regulation in adjuvant-induced arthritic rats. Further investigation supported that astilbin may offer antiarthritis benefits by inhibiting the TLRs-mediated NF-*κ*B signaling. Overall findings suggest that astilbin modulates inflammatory mediators by which GZ-SRG exhibits its therapeutic potential in the treatment of RA disease.

## Figures and Tables

**Table 1:** Primers used for quantitative real-time PCR.

	Forward	Reverse
TNF-*α*	CTACTGAACTTCGGGGTGATCG	GTTGTCTTTGAGATCCATGCCATTG
IL-1*β*	CCCAACTGGTACATCAGCACCTCTC	CTATGTCCCGACCATTGCTG
IL6	GATTGTATGAACAGCGATGATGC	AGAAACGGAACTCCAGAAGACC
GAPDH	TGGAGTCTACTGGCGTCTT	TGTCATATTTCTCGTGGTTCA

**Table 2:** Effects of astilbin (GZ-SRG) on paw thickness in CFA-induced arthritic rats (*n* = 10).

Day	Paw thickness (mm)			
	Control	Model	Astilbin	LEF
d1	3.58 ± 0.09	3.72 ± 0.20	3.72 ± 0.06	3.73 ± 0.06
d7	3.69 ± 0.04	4.16 ± 0.17^##^	3.99 ± 0.24	3.98 ± 0.16^*∗*^
d10	3.70 ± 0.12	5.41 ± 1.21^##^	4.01 ± 0.30^*∗*^	4.03 ± 0.27^*∗∗*^
d13	3.73 ± 0.09	5.98 ± 1.07^##^	4.10 ± 0.26^*∗*^	3.98 ± 0.20^*∗∗*^
d16	3.66 ± 0.04	6.29 ± 1.55^##^	4.03 ± 0.32^*∗∗*^	3.98 ± 0.15^*∗∗*^
d19	3.70 ± 0.09	6.42 ± 1.47^##^	4.14 ± 0.33^*∗∗*^	3.94 ± 0.09^*∗∗*^
d22	3.71 ± 0.07	6.32 ± 2.96^##*δ*^	3.97 ± 0.23^*∗*^	3.92 ± 0.14^*∗*^
d25	3.68 ± 0.12	6.81 ± 2.42^##^	4.19 ± 0.62^*∗*^	3.96 ± 0.18^*∗*^
d28	3.68 ± 0.14	6.79 ± 2.70^##^	3.84 ± 0.14^*∗*^	3.83 ± 0.13^*∗*^

Notes: the development of arthritis was monitored by measuring the paw thickness using a vernier scale. Data represent the mean values of three experiments ±SD. ^##^*p* < 0.01, versus control group; ^*∗*^*p* < 0.05, ^*∗∗*^*p* < 0.01, versus CFA-induced arthritic rat model group; ^*δ*^data from 9 rats after removing one anomalous value.

## Abbreviations

AA: Adjuvant arthritis
CFA: Complete Freund's adjuvant
IKK*β*: Inhibitory protein kappa B kinase beta
IL: Interleukin
LEF: Leflunomide
MYD88: Myeloid differentiation primary response gene 88
NF-*κ*B: Nuclear factor kappa B
RA: Rheumatoid arthritis
SGR: * Smilax glabra* Roxb.
TLR: Toll-like receptor
TNF-*α*: Tumor necrosis factor alpha.
