# Corrigendum to “Astilbin from* Smilax glabra* Roxb. Attenuates Inflammatory Responses in Complete Freund's Adjuvant-Induced Arthritis Rats”

**DOI:** 10.1155/2018/6279328

**Published:** 2018-12-02

**Authors:** Lisha Dong, Jinqiu Zhu, Hongzhi Du, Heng Nong, Xichen He, Xiaoyu Chen

**Affiliations:** ^1^School of Pharmacy, Guiyang College of Traditional Chinese Medicine, Guiyang 550002, China; ^2^Center of Miao Medicine Engineering Technology, Guiyang 550002, China; ^3^Department of Epidemiology and Environmental Health, The State University of New York, Buffalo, NY, USA; ^4^School of Chinese Medicine, Hong Kong Baptist University, Kowloon Tong, Hong Kong

In the article titled “Astilbin from* Smilax glabra* Roxb. Attenuates Inflammatory Responses in Complete Freund's Adjuvant-Induced Arthritis Rats” [1], the affiliation of Dr. Jinqiu Zhu should be “Department of Epidemiology and Environmental Health, The State University of New York, Buffalo, NY, USA” only. The updated authors' list and affiliations are shown above.
